# Research Progress of Heavy-Metal-Free Quantum Dot Light-Emitting Diodes

**DOI:** 10.3390/nano14100832

**Published:** 2024-05-09

**Authors:** Ruiqiang Xu, Shi Lai, Youwei Zhang, Xiaoli Zhang

**Affiliations:** Guangdong Provincial Key Laboratory of Information Photonics Technology, School of Physics and Opto-Electronic Engineering, Guangdong University of Technology, Guangzhou 510006, China; 18475330011@163.com (R.X.); 18565578056@163.com (S.L.); wzhgwb159@163.com (Y.Z.)

**Keywords:** heavy-metal-free quantum dot light-emitting diodes, QD emitter layer, hole transport layer, electron transport layer

## Abstract

At present, heavy-metal-free quantum dot light-emitting diodes (QLEDs) have shown great potential as a research hotspot in the field of optoelectronic devices. This article reviews the research on heavy-metal-free quantum dot (QD) materials and light-emitting diode (LED) devices. In the first section, we discussed the hazards of heavy-metal-containing quantum dots (QDs), such as environmental pollution and human health risks. Next, the main representatives of heavy-metal-free QDs were introduced, such as InP, ZnE (E=S, Se and Te), CuInS_2_, Ag_2_S, and so on. In the next section, we discussed the synthesis methods of heavy-metal-free QDs, including the hot injection (HI) method, the heat up (HU) method, the cation exchange (CE) method, the successful ionic layer adsorption and reaction (SILAR) method, and so on. Finally, important progress in the development of heavy-metal-free QLEDs was summarized in three aspects (QD emitter layer, hole transport layer, and electron transport layer).

## 1. Introduction

In recent years, with the rise in green energy and environmental protection technologies, heavy-metal-free QLEDs have gradually become a research hotspot in the field of optoelectronic devices as a remarkable innovative technology. Traditional QDs containing heavy metals (Cd, Pb, Hg) have received widespread attention due to their excellent optical and electrical properties, as well as the excellent performance of optoelectronic devices based on this material. Although significant achievements have been made in the field of heavy-metal-containing QDs in the past few decades, their commercial applications have been largely limited due to the presence of heavy metal elements harmful to the environment and human health. In this context, heavy-metal-free QDs have become a promising alternative to heavy-metal-containing QDs due to their low toxicity and comparable optical and electrical properties, and have shown great potential in light-emitting diode applications.

So far, the most widely studied semiconductor nanomaterials include cadmium (Cd)-, lead (Pb)-, and mercury (Hg)-based metal chalcogenide QDs. This is because these materials are relatively easy to prepare, their absorption and emission range covers the ultraviolet (UV) to mid-infrared (MIR) region, and their optoelectronic devices exhibit excellent performance based on them. Although dangling bonds may lead to the formation of surface defects, the photoluminescence quantum yields (PLQYs) of QDs can reach up to 70% to nearly 100% by coating the inorganic shell to passivate these surface defects. Therefore, this enables QDs to become luminescent materials for visible and near-infrared (NIR) light-emitting diodes (LEDs) [1,2,3,4]. Although significant progress has been made in the synthesis and application of Cd-, Pb-, and Hg-based chalcogenide QDs, the harm of heavy metals and their corresponding QDs has attracted people’s attention, with the deepening of research on their toxicity mechanisms. The heavy metal elements used in traditional LED preparation not only pose potential hazards to the ecosystem, but also pose challenging environmental issues in waste disposal. Therefore, the research and application of heavy-metal-free QLEDs has become one of the common efforts of scientists. The currently studied heavy-metal-free QDs include InP [5], ZnE (E=S, Se, and Te) [6,7], CuInE_2_ (CIE) [8,9], Ag_2_E [10], and AgInE_2_ (AIE) [11], as well as Mn^2+^ or Cu^+^-doped ZnSe [12], InP [13], and ZIE [14,15], aiming to substitute heavy-metal-containing QDs. Compared with heavy-metal-containing QDs, heavy-metal-free QDs have similar optical properties, making them promising materials for efficient LED applications [7,16]. In addition, due to the fact that heavy-metal-free QDs do not contain heavy metal elements, they have lower toxicity compared to heavy-metal-containing QDs [17,18,19,20]. Under this trend, research into heavy-metal-free QLEDs can not only contribute to sustainable development and green energy, but also provide us with a more environmentally friendly and sustainable choice of optoelectronic devices, driving the continuous advancement of technology in this field.

In this article, firstly, the hazards of heavy-metal-containing QDs are explained, and the main representatives of heavy-metal-free QDs are introduced. Further in-depth research was conducted on the synthesis methods of heavy-metal-free QDs and the device level of QLEDs, in order to present readers with a comprehensive and profound understanding. Finally, we will present our views on potential challenges and provide prospects in this dynamic research field. Through in-depth research and exploration, we are expecting to better understand and promote the application of heavy-metal-free QLEDs in future lighting and display technologies.

## 2. The Importance of Heavy-Metal-Free QDs

### 2.1. Hazards with Heavy-Metal-Containing QDs

#### 2.1.1. Environmental Pollution

The harm of heavy-metal-containing QDs is closely related to environmental pollution, as these heavy metals not only pollute the environment but also enter the human body through the food chain, causing adverse effects on human health and ecosystems. This issue involves the transportation and migration of heavy metals in the environment. The migration of heavy metals is a complex process in which they enter ecosystems through various pathways. In addition to direct contact with heavy metals, there are also absorption pathways in food, water, and the atmosphere [21] which are caused by the transportation of heavy metals in the environment. Taking Hg as an example, the migration process of other heavy metals such as Pb and Cd is similar to that of Hg.

Every year, a large amount of Hg is emitted into the atmosphere, including through the recycling of anthropogenic Hg (60%), anthropogenic sources (30%), and natural sources (10%) [22]. This Hg mainly comes from industrial activities such as coal-fired power plants, manual and small-scale gold mining, primary metal production, and non-ferrous metal smelting. Once Hg enters the environment, it can diffuse and transport through media such as the atmosphere, water, and soil. Among them, atmospheric motion is the main diffusion medium [23], causing most of the Hg in the atmosphere to enter water and land surfaces. In the environment, inorganic Hg may be methylated into highly toxic methylmercury, which is bioaccumulated through the food chain [24], such as through fish and rice rich in methylmercury [25,26,27,28]. For example, by consuming algae and plankton containing methylmercury, the concentration of methylmercury in fish may reach thousands of times the original concentration. The result of this bioaccumulation is that top consumers in the food chain, such as humans, may have ingested high concentrations of methylmercury. The absorption of highly toxic methylmercury by humans poses a serious threat to health, which will be further elaborated on in the following section.

This environmental pollution issue is not limited to Hg, but also applies to other materials containing heavy metals, including heavy-metal-containing QDs. This highlights the importance of developing heavy-metal-free QDs as environmentally friendly alternative materials to reduce the harm of environmental pollution to ecosystems and human health.

#### 2.1.2. Human Health Risks

The use of some heavy metals has a long history. In ancient Rome, Pb and Hg were widely used for various purposes, such as sweetening old wine and soothing teething pains in infants [29]. However, heavy-metal-containing QDs and the heavy metals themselves pose serious risks to human health. Modern research indicates that the main target organ of Hg is the brain, which can lead to neurological dysfunction, such as Minamata disease, discovered in the 1950s [29]. In addition, Hg has a strong binding ability to freely obtained mercaptans, thus disrupting the cellular structure. The toxicity of Pb is mainly produced through ion mechanisms, where the ionic form of Pb^2+^ can replace cations in other organisms, such as Mg^2+^, Fe^2+^, Ca^2+^, Na^+^, etc., interfering with cellular metabolism and leading to various diseases in the nervous, reproductive, renal, and blood systems. The industrial use history of Cd is relatively short, but its toxicity is still significant, especially in terms of kidney damage. Cd accumulates in the kidneys and has adverse effects on the activity of metalloenzymes, affecting protein metabolism and intracellular calcium absorption [30]. In addition, Cd may also have side effects on neurotransmitter metabolism, blood glucose homeostasis, and the normal function of alkaline phosphatase in the central nervous system [31].

In this context, heavy-metal-containing QDs have also attracted attention. Research has shown that heavy-metal-containing QDs may be toxic to cells under certain conditions, and even under long-term exposure and UV irradiation, high QD concentrations cannot completely eliminate toxicity [32,33,34]. In addition, Cd-based QDs can induce reactive oxygen species (ROS), disrupt oxidative balance, and lead to oxidative stress, cell apoptosis, and DNA damage [35,36,37].

In summary, heavy-metal-containing QDs and the toxicity mechanisms of heavy metals themselves share similarities and pose potential threats to human health. Therefore, researchers and industry need to pay special attention to these risks and actively seek safer alternative materials to reduce potential hazards to human health. Research in this field also needs to delve deeper into the effects of surface modification and exposure conditions of QDs on toxicity to ensure their safety in applications.

### 2.2. Main Representatives of Heavy-Metal-Free QDs

Due to the widespread application potential and excellent safety of heavy-metal-free QDs, interest in their development is increasing. In the past few decades, various heavy-metal-free QDs have been successfully developed, such as InP [13,16,38,39,40], ZnE (E=S, Se and Te) [6,7,41], CuInS_2_ [8,42], Ag_2_S [43], and so on [14,44,45,46], as shown in Table 1. These non-toxic or less-toxic QDs are considered promising candidate materials to replace toxic heavy-metal-containing QDs.

Among them, InP QDs are widely used in the study of heavy-metal-free QDs. They have unique characteristics such as direct bandgap, high electron mobility, and large bandgap value, making them ideal semiconductor material for high-performance optoelectronic devices [47]. However, it is relatively difficult to prepare InP QDs with precise size control, and they are sensitive to oxidation and are likely to convert into In_2_O_3_ when exposed to air [48]. Therefore, InP QDs need to be synthesized under strict airless conditions. To improve stability and suppress surface defects, it is usually necessary to add an appropriate shell layer to the core of InP QDs. By using a wider bandgap shell to restrict the wave function of electron-hole pairs within the InP core region, quantum yield (QYs) can be significantly improved and photobleaching and photointermittency can be suppressed [49].

In addition to InP QDs, Zn-based semiconductor nanomaterials have also attracted widespread attention, especially QD materials containing ZnS and ZnSe. These materials have the advantage of adjustable absorption and emission wavelengths, giving them potential Cd-free blue luminescent properties in various applications such as blue LEDs, sensors, catalysis, etc. [7,50]. In addition, ZnS and ZnSe are considered promising materials for inorganic passivation shells which can improve the stability and emission performance of core QDs [51].

Copper chalcogenide nanomaterials have attracted much attention due to their low toxicity, low cost, and environmental compatibility [52,53]. They have shown enormous potential in fields such as lighting, photocatalysis, and biomedical imaging [54,55,56]. At present, Cu_22−x_S and Cu_2−x_Se are the most studied binary copper chalcogenide compounds. For ternary copper chalcogenide compounds, CuInS_2_ and CuInSe_2_ have the potential to replace Cd and Pb chalcogenide QD materials in applications such as LED [42,57] and optical imaging [58] due to their tunable n-type or p-type conductivity.

Silver chalcogenide QDs are considered promising photovoltaic materials, especially in catalysis and solar cells, due to their relatively narrow bandgap and tunable emission wavelength [59,60,61]. In addition, their low solubility indicates that Ag^+^ ions are not easily released in biological systems, and therefore have broad application prospects in the fields of biology and biomedical science [62,63].

The application of Pb-free metal halide perovskite QDs in the field of optoelectronics has also received much attention. Among them, Sn-based perovskite materials are considered promising candidate materials due to their similar properties to Pb-based perovskite and excellent performance in optoelectronic devices [64]. Although Ge-based perovskite materials also have similar properties, their performance in optoelectronic devices is poorer compared to Sn-based perovskite materials [65].

In summary, heavy-metal-free QD materials have shown remarkable performance and broad application prospects in various fields, which is of great significance for replacing toxic heavy-metal-containing materials.

## 3. The Synthesis Methods of Heavy-Metal-Free Nanostructures

The synthesis of heavy-metal-free QDs involves multiple complex steps and mechanisms, among which the formation of one-dimensional nanostructures is crucial. When synthesizing one-dimensional slender nanoparticles, aspect ratios are usually used to distinguish different structures, such as nanowires (NWs) and nanorods (NRs). To achieve this unique one-dimensional morphology, four growth mechanisms were employed, including solution–liquid–solid (SLS), oriented attachment, template-assisted growth, and ripening. For example, inspired by a vapor–liquid–solid (VLS) method [66], Trentler et al. [67] developed the SLS method. This method has been successfully applied to the growth of NRs, achieving precise control of the diameter and length of Bi-InP heterostructure NRs by controlling the Bi nanocrystalline (NC) catalyst [68]. Oriented attachment is a spontaneous self-organizing process used to prepare single crystal NRs with a common crystal orientation. Pacholski et al. [69] achieved the oriented attachment of ZnO NRs by heating the prepared quasi-spherical ZnO NRs in alkaline water, resulting in the formation of single crystal wurtzite ZnO NRs. In addition, during the synthesis of ZnS and ZnSe, 1D ZnS NCs and ZnSe NCs can also be formed along specific directions through oriented attachment technology [70,71].

In addition to the NWs and NRs mentioned above, there are also their one-dimensional morphology nanotubes, and their preparation methods involve various approaches such as template-assisted growth, chemical conversion, and etching. The template-assisted growth method has been widely used in the preparation of various semiconductor nanotubes, by the epitaxial growth of semiconductor nanotubes on NR/NW templates, and then the removal of the templates to form nanotubes. Taking GaN nanotubes as an example, a ZnO NWs template is used in a reaction tube containing trimethylgallium and ammonia, and argon or nitrogen is used as the carrier gas to form GaN nanotubes through a series of steps [72]. Similarly, ZnO nanotube arrays were successfully prepared by pre coating ZnO thin films on silicon wafers, followed by pulsed laser deposition and hydrothermal growth [73]. In addition, the chemical conversion method has also been applied to the preparation of semiconductor nanotubes; for example, by heating ZnO NWs and thioacetamide aqueous solution at 90 °C for 9 h, ZnS nanotubes with a diameter of 70 nm and a wall thickness of 16–20 nm are formed [74].

Similar to one-dimensional NCs, the synthesis of two-dimensional NCs is equally important. Compared to the lateral size, two-dimensional NCs have a smaller thickness morphology. Research has shown that the growth mechanisms of heavy-metal-free two-dimensional nanomaterials include oriented attachment growth and template-assisted growth. Through the oriented attachment growth mechanism, the synthesis of ZnSe nanoplatelets (NPLs) with a thickness of 1.39 nm and a smaller lateral size was successfully achieved [75]. By adjusting the reaction conditions and material concentration, NPLs with atomic flatness were produced, exhibiting ultra-narrow band edge emission peaks. And template-assisted growth has also been used to prepare two-dimensional nanostructures, such as Cu_2−x_S nanosheets formed through synergistic interactions [76,77].

**Figure 1:**
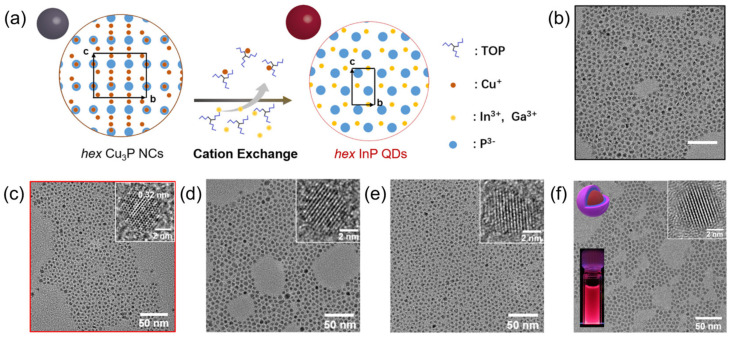
(**a**) Schematic illustration of CE synthesis. The projection of the original unit cell is indicated with a black rectangle in the structure. TEM images of GaP QDs with (**b**) initial exchange and (**c**) two successive exchanges from Cu_3_P-2.25 NCs. The inset shows the HRTEM image of the resultant GaP QDs. (**d**) TEM image of In-rich InGaP alloy QDs. (**e**) TEM image of Ga-rich InGaP alloy QDs. The insets in (**d**,**e**) show corresponding HRTEM images. (**f**) TEM image of InP-2.25/ZnSeS core/shell QDs. The insets show the corresponding HRTEM image and optical photograph under UV illumination. Reproduced from Ref. [40] with permission from the American Chemical Society.

At present, the commonly used methods for synthesizing heavy-metal-free QDs include the HI method, the HU method, the CE method, the SILAR method, and so on. The HI method involves rapidly injecting a hot precursor solution into a reaction mixture at a high reaction temperature. Won et al. [16] synthesized excellent InP/ZnSe/ZnS QDs using this method and applied them to LEDs, with a device performance comparable to that of Cd-based QLEDs. The characteristic of the HU method is the gradual increase in the reaction mixture temperature. Zhang et al. [14] synthesized a series of Cu-doped ZnInS QDs using a one-pot non-injection synthesis method, and the prepared electroluminescent devices had good performance. The CE method involves replacing the cations in the pre synthesized QDs with new cations. Shan et al. [40] synthesized metastable wurtzite mono-disperse InP QDs using Cu_3_P nanocrystals as raw materials through a cation exchange reaction, as shown in Figure 1. The SILAR method is a layer-by-layer deposition technique that involves alternating the adsorption of cations and anions onto the substrate surface, followed by reaction steps. Xie et al. [13] successfully synthesized efficient Cu-doped InP QDs using HI and SILAR methods, and systematically studied the doping process and dopant diffusion during shell epitaxy. Therefore, the above methods provide diverse and controllable pathways for synthesizing various heavy-metal-free QDs and contribute to their advantages in applications such as optics and electronics.

## 4. Overview of the Development in Heavy-Metal-Free QLEDs

In QLEDs, the PLQYs and the charge injection balance of the QD emitter layers are key factors determining device performance. Firstly, in order to achieve efficient PLQYs, key parameters that need to be optimized include size, shape, element composition, core–shell energy-level arrangement, surface ligand thickness, surface/interface defects, and properties. Secondly, optimizing the charge balance requires appropriately reducing the hole/electron injection energy barriers of each layer (emitter layer and hole/electron transport layer) and increasing or synchronizing their charge mobility. At present, the most commonly used heavy-metal-free QD emitter layer material in quantum dot light-emitting diode (QLED) devices is InP QDs. Compared to previous studies on more Cd-based QDs, InP-based QDs generally exhibit a shallower valence band maximum (VBM). Therefore, the hole injection barrier from the hole transport layer to the InP-based QD emission layer is smaller, which is beneficial for hole injection. For ZnO-based electron transport layers, a deeper VBM is advantageous for capturing holes in the QD emitter layer, while a lower conduction band minimum (CBM) suppresses electron injection. Therefore, in order to control the electron injection barrier, various doped ZnO electron transport layers have been proposed [78]. Table 2 describes the characteristics of heavy-metal-free QLEDs mentioned below. The development of heavy-metal-free QLEDs will be summarized in three aspects: the QD emitter layer, hole transport layer, and electron transport layer.

### 4.1. QD-Emitting Layer

In the early development of QLEDs, research mainly focused on QD materials with different compositions such as ZnSe [79], PbS [80], and CdSe [81]. Among them, CdSe-based QDs have received widespread attention and have strong competitiveness in display applications. However, due to the restrictions on harmful substances such as Cd in the RoHS (Restriction of Hazardous Substances) standard issued by the European Union in 2002, this has had a profound impact on the LED device industry and scientific research direction that uses heavy-metal-containing QDs such as Cd. Therefore, research on the emission layer of heavy-metal-free QDs has become an inevitable trend, especially the exploration of alternatives to CdSe QDs, which has become an urgent need. InP QDs have become the most promising candidate in display applications due to their bandgap covering all visible light emission ranges and their high efficiency in direct energy conversion properties. By carefully designing the size, shape, and surface properties of heavy-metal-free QDs, emission spectra can be controlled to meet the display needs of different colors.

In 2002, Battaglia et al. [82] proposed a method for the rapid synthesis of InP QDs using indium acetate and trimethylsilylphosphine precursors in a 1-octadecene solvent at 270 °C. Subsequently, researchers continuously improved the structure of InP QDs, including the design of core/shell structures, to enhance their luminescent properties and performance in QLED applications. For example, Lim et al. [83] synthesized a stability-enhanced composite with a gradient shell composition InP@ZnSeS QDs and demonstrated colloidal green luminescent InP-based QLEDs. Cao et al. [84] synthesized large-sized InP/ZnSe/ZnS QDs using a layer-by-layer shell growth strategy, with a PLQY of up to 73%. The prepared red QLED device achieved an external quantum efficiency (EQE) of 6.6%. In addition, researchers have continuously improved the performance of InP QDs by introducing GaP interface layers and optimizing the structure of the shell. For example, the green QLED based on the InP/GaP/ZnS//ZnS core–shell structure prepared by Zhang et al. [85] achieved peak EQE and current efficiency of 6.3% and 13.7 cd A^−1^, respectively. In this development process, the design principle of the InP/ZnSe_x_S_1−x_/ZnS multi shell has been comprehensively studied, and the structure of the multi-shell composite has been designed through engineering to better meet the needs of practical applications [86]. Although some challenges still need to be overcome in these processes, such as the inherent weakness of oxidation, the performance of InP QDs as substitutes for heavy-metal-containing QDs in the emission layer is already encouraging. In addition, for commercial considerations, perovskite QDs have also received much attention. But typically, perovskite QD materials use heavy metal elements, which are highly anticipated due to their simple preparation, inherent defect-free energy levels, and high quantum efficiency [87,88].

**Figure 2:**
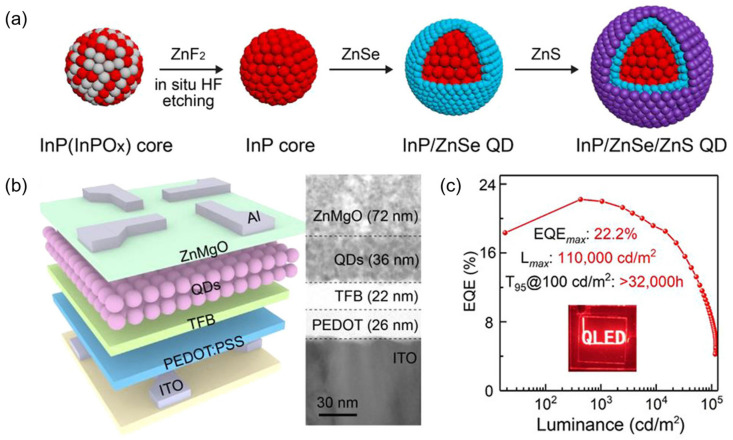
(**a**) Scheme of the synthesis of highly luminescent InP/ZnSe/ZnS QDs with ZnF_2_. (**b**) Scheme of the QLEDs and cross-sectional TEM image of the QLED with ∼4 monolayers of QDs. (**c**) EQE–luminance profile. Inset, the photograph of text-patterned QLED. Reproduced from Ref. [89] with permission from the American Chemical Society.

**Figure 3:**
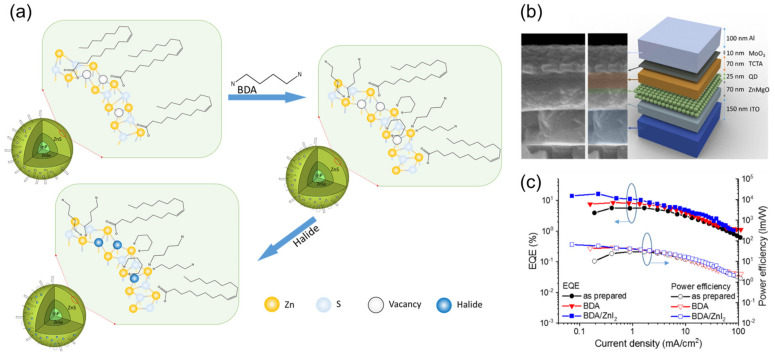
(**a**) The schematic diagram of InP GQDs passivated by the synergistic effect of BDA combined with zinc halides. (**b**) The SEM image of the interlayer cross section and corresponding device structure. (**c**) EQE (left axis) and power efficiency (right axis) versus current density profiles. Reproduced from Ref. [90] with permission from Springer Nature.

In recent years, the development of heavy-metal-free QLEDs has been rapid, and the efficiency of red and green light QLED devices has achieved certain breakthroughs. For example, Won et al. [16] introduced HF etching to oxidize the InP core surface during the initial ZnSe shell growth process and grew it at a high temperature, at 340 °C, to obtain InP-based QDs with fewer defects. The maximum EQE of the optimized InP/ZnSe/ZnS red QLED can reach 21.4%, and its performance can be comparable to the state-of-the-art Cd-based QLED. Li et al. [89] used inorganic salt ZnF_2_ to react gently with carboxylic acid at high temperatures, thereby eliminating surface oxide impurities and promoting the growth of epitaxial shells. The resulting InP/ZnSe/ZnS QDs have better thermal stability. As shown in Figure 2, QLEDs based on such QDs can achieve a maximum EQE of 22.2%. In addition, Chao et al. [90] modified the emission layer of InP QDs by passivating with different alkyl diamines and zinc halides, balancing electron and hole mobility. The maximum EQE of the green InP QLED prepared can reach 16.3%, approaching the theoretical limit of the currently prepared InP green QDs (GQDs), as shown in Figure 3.

**Figure 4:**
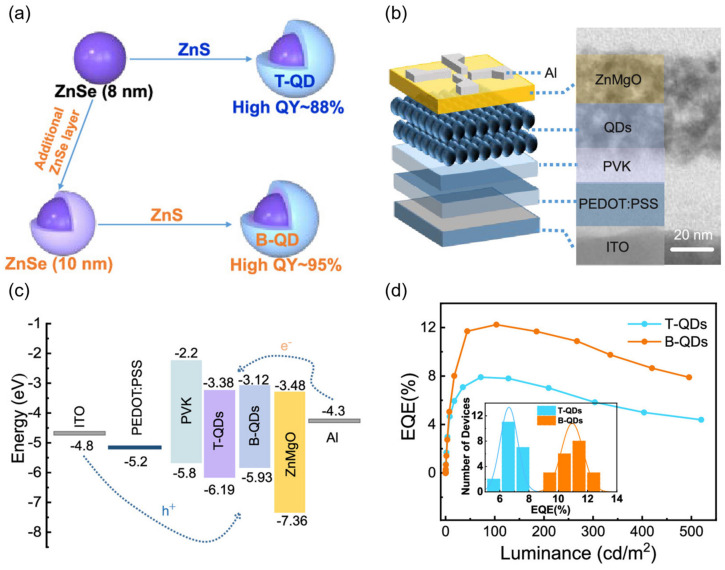
(**a**) Schematic illustration of the controlled synthesis of two types of ZnSe/ZnS core/shell QDs. Traditional ZnSe/ZnS QDs (T-QDs) were obtained by growing a ZnS shell (4 MLs) on the ~8.0 nm ZnSe core. Bulk-like ZnSe/ZnS QDs (B-QDs) were obtained by growing a ZnS shell (4 MLs) on the ∼10.0 nm ZnSe core. (**b**) Scheme and cross-sectional TEM image of QLED with B-QDs as EML. (**c**) Band energy level diagram of QLED device. (**d**) EQE versus the luminance for typical B-QDs- and T-QDs-based QLEDs. Insert, histograms of the peak current efficiency (CE) and EQE for the two types of QLEDs. Reproduced from Ref. [91] with permission from the American Chemical Society.

For the less stable heavy-metal-free blue light QLED, its device efficiency and service life have also been improved to a certain extent. For example, Gao et al. [91] synthesized blue light QDs by growing thin shell layers of ZnS on ZnSe nuclei larger than the Bohr volume diameter, and used them to prepare LEDs with an EQE of 12.2% and a relatively long working life, as shown in Figure 4. These synthesized high-quality QDs reduce the energy level difference between QD and adjacent layers in LED and effectively improve charge transfer. In addition, Kim et al. [7] are committed to developing efficient and stable Cd-free blue-light-emitting devices. By using ZnCl_2_ for chlorination passivation and a double-QD-emitting layer structure, the EQE of the blue QLED prepared is close to the theoretical limit (20.2%) and greatly improves its service life.

In summary, the development of heavy-metal-free QD emission layers aims to achieve more efficient and stable luminescence, in order to meet the requirements of commercial displays for high efficiency and long lifespan. With in-depth research on these emission layer materials and structures, heavy-metal-free QLED devices will continue to move towards a more mature and commercialized stage. In this exploration process, alternatives to CdSe QDs, especially new heavy-metal-free materials such as InP QDs, will become a key direction for future development to address the dual challenges of environmental protection and performance.

### 4.2. Hole Transport Layer

Under normal circumstances, the charge transfer rate of organic hole transport materials in QLED is slower than that of QDs or inorganic electron transport materials, leading to charge imbalance. In addition, the energy barrier for hole injection from the hole transport layer to the QD layer is high. To address this issue, various methods have been adopted to increase conductivity and control the energy levels of the hole transport layer.

**Figure 5:**
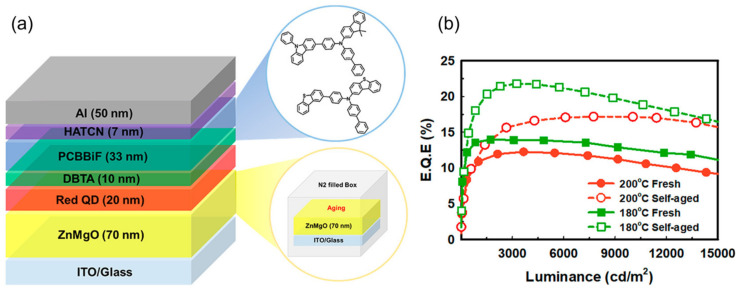
(**a**) Device structure of the inverted red QLEDs. (**b**) External quantum efficiency versus luminance properties of QLEDs. Reproduced from Ref. [94] with permission from the American Chemical Society.

For QLEDs with inverted structures, the available hole transport materials are relatively limited, such as 4,4-N,N-dicarbazole-biphenyl (CBP) [92], 2,2′,7,7′-tetrakis [N-naphthalenyl (phenyl) amino]-9,9-spirobifluorene (spiro-2NPB) [1], etc. Lee et al. [93] achieved high-performance green and red inverted top emitting InP/ZnSeS QLEDs by introducing CzSi as a hole suppression interlayer to control the charge balance. Yeom et al. [94] successfully prepared an efficient and stable InP-based inverted red QLED by using a new hole transport material, DBTA, and an optimized sol-gel ZnMgO layer, with the maximum EQE of 21.8%, as shown in Figure 5. The new hole transport material, DBTA, has high hole mobility and deep HOMO energy levels, allowing for the faster injection of holes into InP QDs.

**Figure 6:**
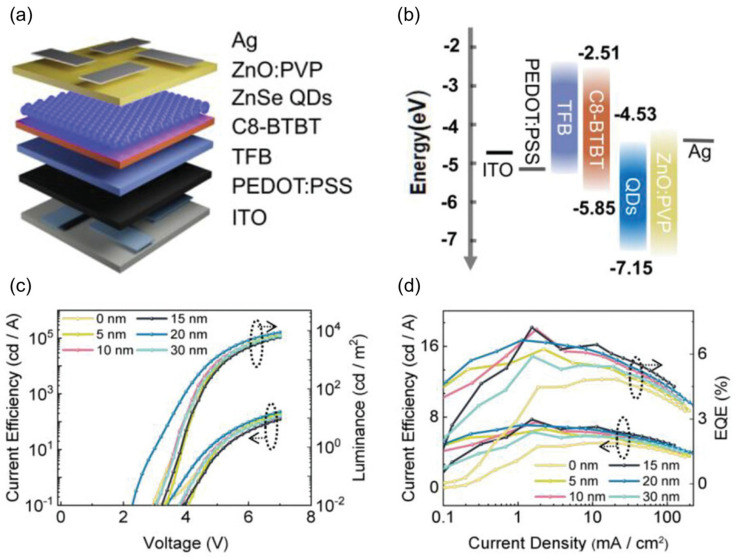
(**a**) Schematic of the device structure. (**b**) QLED schematic of energy band alignment. (**c**) J–V–L and (**d**) CE–V–L for QLEDs with different C8-BTBT thicknesses. Reproduced from Ref. [99] with permission from John Wiley and Sons.

For QLEDs with an upright structure, there are more available hole transport materials, such as polystyrene N,N′-diphenyl-N,N′-bis (4-n-butylphenyl)-(1,1′-diphhenyl)-4,4′-diamine perfluorocyclobutane (PS-TPD-PFCB) [95], [N,N′-bis (1-naphthyl)-N,N′-diphhenyl-1,1′-diphhenyl-4,4′-diamine] functionalized with two styryl groups s (2-TPD), 4,4′,4′′-tri(N-carbazolyl) triphenylamine bis (vinylbenzyl ether) (TCTA-VB) [96], cross linked poly (9,9-dioctylfluorene co N-(4-(3-methylpropyl)) diphenylamine (TFB) [97], etc. At present, the most commonly used hole transport materials are PVK [91,98] and TFB [7,16], and of these PVK has a deeper HOMO energy level but a lower hole mobility. TFB has become the most popular hole transport material for QLED due to its high mobility. In addition, the study of multi-hole transport layer structures has also become one of the exploration directions. For example, Luo et al. [99] prepared a high-performance Cd-free blue ZnSe/ZnS QLEDs by using a TFB/C8-BTBT dual hole transport layer, as shown in Figure 6; its maximum EQE was 7.23%, which is nearly 150% higher than traditional devices based on a single hole transport layer. Research has shown that introducing a TFB/C8-BTBT dual hole transport layer can effectively reduce the charge accumulation between the HTL and QD emission layer, and improve hole injection in Cd-free blue light QLEDs.

In addition to organic materials, some inorganic materials such as NiO can be introduced as hole injection layers. NiO has a more suitable band shift compared to organic hole transport materials, and its hydrophilicity makes it convenient to prepare as a separate layer next to the QD emitter layer [100]. Other inorganic hole transport materials, such as Cl passivated tungsten phase (Cl-TPA), have also shown potential advantages by exhibiting shallow HOMO levels, which are beneficial for hole injection [101].

Although organic hole transport materials perform well in QLEDs, some challenges still exist, such as the poor solubility resistance of polymers. To address this issue, the development of new hole transport materials has become crucial. Overall, the introduction of inorganic materials such as NiO and Cl-TPA as hole transport layers has shown potential advantages in QLED. However, further optimization of material composition and process conditions is still needed to improve the operational stability of QLEDs. Secondly, considering that the degradation of organic materials at the interface with QDs may result in nonradiative recombination centers, future research may focus on developing more high-performance inorganic hole transport materials suitable for QLEDs.

### 4.3. Electron Transport Layer

In addition to the QD emission layer and hole transport layer mentioned above, the electron transport layer also has a crucial impact on the efficiency and working life of QLED devices. In the early development of QLEDs, due to the much higher electron affinity of QDs compared to semiconductor organic polymers [102], researchers focused more on how to improve hole injection performance. Some early QLEDs did not even have an electron transport layer, but only a thin QD film sandwiched between the electrodes. This will lead to the behavior of space charge limiting current, resulting in lower electron mobility.

**Figure 7:**
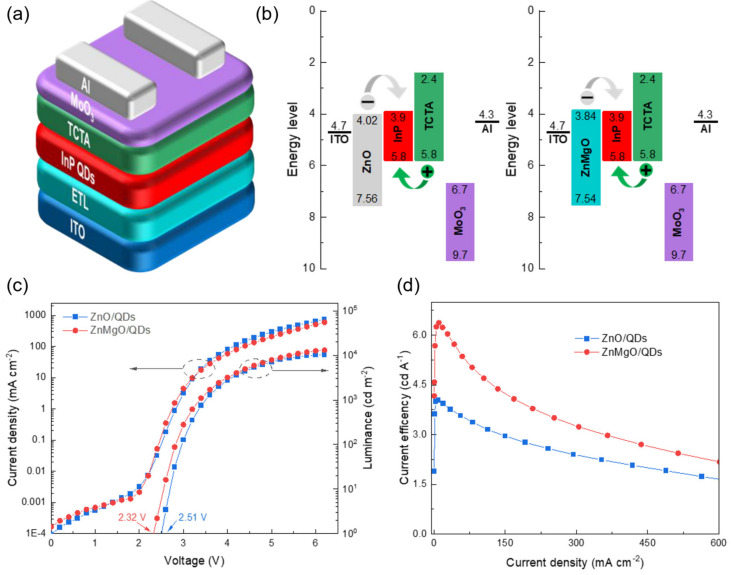
(**a**) Schematic diagram showing the structure of the device. (**b**) Band diagrams of the QLED with ZnO ETL and ZnMgO ETL. (**c**) J-V-L curves of inverted InP QLED with different ETL. (**d**) The dependence of current efficiency on current density. Reproduced from Ref. [103] with permission from MDPI.

**Figure 8:**
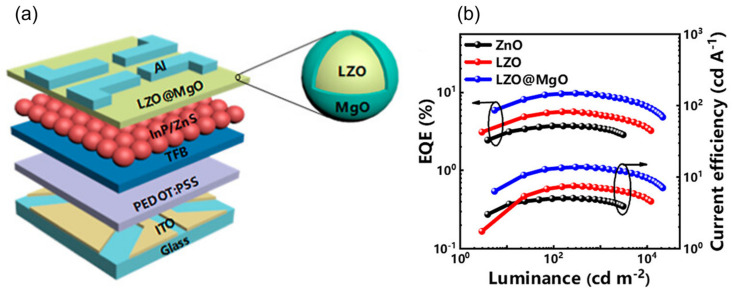
(**a**) The optimized device structure of QLEDs. (**b**) EQE–luminance–current efficiency characteristics of QLEDs. Reproduced from Ref. [105] with permission from the American Chemical Society.

At present, the rapid development of research has led to the proposal and application of many electron transfer materials in the preparation of QLED devices, such as ZnO, ZnMgO, etc. They exhibit optoelectronic properties similar to QDs in terms of size/shape, film morphology, organic impurities, etc. For the improvement of electron injection performance, electron transport layers with larger bandgaps than QD emitters can also be utilized, such as TPBi, BCP, etc. In addition to the proposal of new electronic transport materials, researchers have also attempted different strategies, such as doping and adding intermediate layers. Zhang et al. [103] prepared an environmentally friendly InP-based inverted red QLED by using Mg-doped ZnO as the electron transport layer (ETL). The study showed that the conduction band of Mg-doped ZnO shifted towards a position that better matched the InP QDs, effectively enhancing the performance of QLEDs, as shown in Figure 7. Mude et al. [104] used Ni-doped ZnO as the electron transport layer and introduced a ZnS interlayer at the ETL/QD interface to prepare a high-performance InP-based inverted red Cd-free QLED device, with a maximum EQE of 10.6%. By utilizing Ni doping, the conductivity and minimum conduction band of ZnO can be adjusted, which helps to enhance the charge balance in QLED devices. And the introduction of a ZnS interlayer at the ETL/QD interface further suppresses exciton quenching. Ning et al. [105] reported a strategy based on Li doping and MgO shell coating, which effectively improved the performance of InP-based QLEDs by adjusting the defect state of ZnO, as shown in Figure 8. Research has shown that Li doping passivates the intrinsic defect states of ZnO NPs, improving electron mobility, while the MgO shell layer passivates the surface oxygen defects of ZnO NPs, thereby reducing exciton quenching at the ZnO/QD interface.

In addition, the stability of organic electron transfer materials has always been a major concern, such as the poor thermal stability of Alq_3_ and the complex preparation process of its films, resulting in poor carrier transport capacity. And metal oxides (such as ZnO, TiO_2_, etc.) have better thermal stability than some commonly used organic materials, so they are widely used in the study of QLEDs. At present, ZnO is one of the most popular electron transfer materials, and has the characteristics of good chemical and thermal stability, fast electron transfer rate, etc. It can effectively improve the injection efficiency of electrons and make devices exhibit high environmental stability. By adjusting the composition and structure of the electron transport layer, it is also possible to effectively improve the electron transfer rate and control energy levels, thereby improving device performance. For QLEDs with optimized full solution treatment, researchers have attempted different strategies, including doping, size control, and the addition of intermediate layers. Doping the ZnO electron transport layer can effectively regulate charge balance, while size control and the introduction of the intermediate layer also have a significant impact on electro-optical performance. For example, Guo et al. [106] prepared an all-solution processed green InP QLED, which is achieved by an electron transport layer of In-doped ZnO nanoparticles. This ETL can not only improve the charge balance by preventing a large amount of electron injection, but also suppress exciton quenching generated by the InP QD emission layer, thereby improving device performance.

**Table 2:** Overview of the characteristics for heavy-metal-free QLEDs.

QDs	LED Structure ^1^	EQE (%)	Turn-On Voltage (V)	Max. lum (cd/m^2^)	Peak CE ^2^ (cd/A)	Lifetime	Year	Ref.
InP/ZnSe/ZnS	ZnO/QDs/CBP/HAT-CN/Al	6.6	2	1600	13.6		2018	[84]
InP/GaP/ZnS//ZnS	PEDOT:PSS/TFB/QDs/ZnO/Al	6.3	2.98	2938	13.7		2019	[85]
InP/ZnSe/ZnS	PEDOT:PSS/TFB/QDs/ZnMgO/Al	21.4	1.8	100,000		4300 h ^3^	2019	[16]
InP/ZnSe/ZnS	PEDOT:PSS/TFB/QDs/ZnMgO/Al	22.2	1.87	110,000		>32,000 h ^4^	2022	[89]
InP/ZnSe/ZnS	ZnMgO/QDs/TCTA/MoO_3_/Al	16.3	2.2	12,646.3	57.5		2021	[90]
ZnSe/ZnS	PEDOT:PSS/PVK/QDs/ZnMgO/Al	12.2	4.1	1055	1.7	237 h ^5^	2021	[91]
ZnTeSe/ZnSe/ZnS	PEDOT:PSS/TFB/QD-Cl/ZnMgO/Al	20.2		88,900		15,850 h ^5^	2020	[7]
InP/ZnSe/ZnS	ZnMgO/QDs/DBTA/PCBBiF/HATCN/Al	21.8	3.5	23,300	23.4	72,848 h ^5^	2020	[94]
ZnSe/ZnS	PEDOT:PSS/TFB/C8-BTBT/QDs/ZnO:PVP/Ag	7.23			7.73		2023	[99]
InP	ZnMgO/QDs/TCTA/MoO_3_/Al	~6.4	2.32	13,000	6.38		2021	[103]
InP/ZnSe/ZnS	NiZnO/QDs/DBTA/PCBBiF/HATCN/Al	10.6			10.4	164,048 h ^5^	2023	[104]
InP/ZnS	PEDOT:PSS/TFB/QDs/LZO@MgO/Al	9.7	2.1	22,200	13.8		2023	[105]
InP	PEDOT:PSS/TFB/QDs/ZnO/Al	5.42	2.2	1692	21.22	25 h ^6^	2021	[106]

^1^ Anode structures of ITO (indium tin oxide) are omitted. ^2^ Peak current efficiency (CE). ^3^ Time for 25% decrease at 1000 nit. ^4^ Time for 5% decrease at 100 nit. ^5^ Time for 50% decrease at 100 nit. ^6^ Time for 50% decrease at 400 nit.

## 5. Conclusions and Outlook

In summary, this article delves into the importance of heavy-metal-free QLEDs, summarizes the hazards of heavy-metal-containing QDs, including environmental pollution and human health risks, and emphasizes the necessity of heavy-metal-free QDs. In addition, the main representatives of heavy-metal-free QDs are listed to lay the groundwork for subsequent content. In terms of synthesis methods, various common preparation techniques have been proposed, providing reference for further research. A review of the development of heavy-metal-free QLEDs is presented, with detailed descriptions of their key components. Blue QLEDs, especially heavy-metal-free blue QLEDs, are a hinderance to the overall development of QLEDs due to their inferior performance, including poor efficiency and instable activity. The roles of the QD emission layer, hole transport layer, and electron transport layer in devices have been fully elucidated. Although QLEDs are currently developing rapidly, there are still some key challenges that need to be addressed, such as the stability issue of Cd-free blue QLEDs. Therefore, future research can focus on optimizing synthesis methods of QDs and improving the structural design of QLEDs, thereby achieving more efficient luminescent performance and application promotion. The development prospects of heavy-metal-free QDs are broad, which will bring significant breakthroughs in environmental protection and green energy fields.

## Figures and Tables

**Table 1:** Overview of the synthesis methods and applications for representative heavy-metal-free QDs.

Material	Wavelength (nm)	FWHM (nm)	Method	Applications	References
InP QDs	490–610	64	HI	Visible light emission	[38]
Cu-doped InP QDs	630–1100		HI and SILAR	Visible and NIR light emission	[13]
InP/ZnSe/ZnS QDs	630	35	HI	Blue, green, and red LEDs	[16]
InP/ZnSe/ZnS QDs	618	42	HI	Red LEDs	[39]
InP/ZnSeS and InGaP QDs	674–754	38–50	CE	Visible emission	[40]
ZnS and ZnSe QDs		10–12 (ZnS) 14 (ZnSe)	HI	UV and blue light emission	[6]
Cu- or Mn-doped ZnSe QDs	470–550 (Cu) 575–595 (Mn)		HI	Visible light emission	[41]
ZnTeSe/ZnSe/ZnS QDs	457	36	HI	Blue LEDs	[7]
CuInS_2_/ZnS core/shell QDs	500–950		HI	Visible light emission	[8]
CuInS_2_/ZnS/ZnS core/shell/shell QDs	559	101.9	HU	White LEDs	[42]
Ag_2_S QDs	1058	21	HU	NIR light emission	[43]
AgAuSe QDs	820–1170	90	HI and CE	NIR light emission	[44]
Cu-doped ZnInS QDs	450–810		HU	Red LEDs	[14]
Cu, Mn co-doped ZnInS/ZnS QDs		190	HU	White LEDs	[45]
ZnAgInS/ZnInS/ZnS core/shell/shell QDs	501	>89	HI	White LEDs	[46]
